# Spatial Patterns and Drivers of SME Digitalisation

**DOI:** 10.1007/s13132-023-01257-1

**Published:** 2023-04-22

**Authors:** Adelheid Holl, Ruth Rama

**Affiliations:** 1 Institute of Public Goods and Policies (IPP), CSIC–Consejo Superior de Investigaciones Científicas, C/Albasanz, 26-28, 28037 Madrid, Spain; 2 Institute of Economics, Geography and Demography (IEGD), CSIC–Consejo Superior de Investigaciones Científicas, C/Albasanz, 26-28, 28037 Madrid, Spain

**Keywords:** SMEs, Digitalisation, Technology adoption, Location, Rural areas

## Abstract

**Abstract:**

Digital transformation plays an increasingly important role in the growth and competitiveness of small and medium-sized enterprises (SMEs), yet little is known regarding spatial inequalities in their adoption of advanced digital technologies. Using recent data from the Flash Eurobarometer 486, we study the spatial patterns of drivers for the implementation of new digital technologies in SMEs in Europe. In our analysis, the focus is on the possible influence of location. Considerable heterogeneity of SMEs is found in their propensity to adopt advanced digital technologies related to the strength of the local business environment and to the urban/rural hierarchy.

**Plain English Summary: European SMEs and Digitalisation:**

The adoption of digital technologies favours the competitiveness, resilience, and internationalisation of firms, but SMEs, which form the backbone of the EU economy, are lagging behind. A recent survey reveals that location greatly influences the probability that European SMEs adopt digital technology. Rural and small-town SMEs are less likely to be adopters, even when country, sector, and firm-specific characteristics are taken into account. However, good business environments always encourage the adoption of digitalisation technologies, whatever the geographic location of an SME. Innovators tend to be adopters, especially when they employ green innovation or management innovation. Larger SMEs, companies that are part of a business group, grow more rapidly, and/or export, are all more likely to adopt digital technologies. Policy-makers need to contemplate the urban/rural-divide and promote strong business environments in all types of locations. Public encouragement towards innovation is likely to indirectly promote easier access to digital technologies.

## Introduction

Digitalisation plays an increasingly important role in the growth and competitiveness of firms. Digitalisation is considered one of the main drivers for advanced manufacturing (Chirumalla, 2021), and it is also seen as a catalyst in providing strategic advantages to firms. The adoption of digital technology appears to make firms and, specifically, small business more resilient (European Investment Bank, 2021), and it has also been related to regional resilience (Reveiu et al., 2022). Digitalisation is part of the Fourth Industrial Revolution or what has been termed Industry 4.0. Industry 4.0 constitutes a new productive paradigm that is based on digital transformation. It is characterised by the combination and integration of different digital technologies (DT) with physical production processes, products, and services (Ustundag and Cevikcan, 2018). Digital technology also appears to facilitate the adoption of other types of innovation, such as logistics innovation (Holl & Mariotti, 2021).

Digital transition constitutes a key priority in the EU policy agenda. Digitalisation ranks high in the recent Recovery and Resilience Program which stipulates that Member States need to allocate at least 20% of the total planned budget of 723.8 billion euros to digital transition. The recently adopted Digital Europe Programme (2021–2027), with a planned budget of 7.5 billion euros, constitutes a further important financial instrument dedicated specifically to supporting the digital transition in the EU. The Digital Innovation Hub Programme of the EU is designed for the promotion of the transition into Industry 4.0 in European regions (Hervas-Oliver et al., 2021). In the last few years, the EU has also launched several programmes towards, specifically, the development and digitalisation of rural areas, including precision farming and digital platforms for e-learning, e-health, and e-administration (Zavratnik et al., 2018).

Small and medium-sized enterprises play a crucial role in digital transformation and the transition towards Industry 4.0 because they represent the vast majority of businesses and form the backbone of most economies (European Commission, 2020). However, as highlighted by the Digital Transformation Scoreboard 2018 (Probst et al., 2018), many SMEs may face a wide range of barriers for the adoption of new DT. In innovation adoption, SMEs tend to lag behind (European Investment Bank, 2021), especially regarding technologies of a more advanced and complex nature (Holl et al., 2013; Holl & Mariotti, 2021; Buer et al., 2021; Hizam-Hanafiah & Soomro, 2021). At the same time, recent studies have observed an increasing concentration of innovative activity in large firms (Rammer & Schubert, 2018), and this can present a challenge for wider knowledge diffusion.

Nevertheless, even the adoption of low-cost digital technologies can make a positive difference for SMEs, especially those located in rural and peri-urban areas (Michel-Villareal et al., 2021; Norris, 2020; Gavrila Gavrila & de Lucas Ancillo, 2021). Adoption of DT has been found to enhance the productivity and the export orientation of SMEs (Hwang & Kim, 2021; Hervé et al., 2021) in addition to contributing towards the organisation of complex networks (Aaldering & Song, 2021). While large firms enjoy an advantage in operating over larger geographical distances due to their greater human and organisational resources, digitalisation can help SMEs overcome spatial distances and establish and manage linkages of a more geographically extensive nature (Holl and Rama, 2009) and access information, services, and resources of a higher quality located elsewhere (Norris, 2020; Bánhidi, 2021). In this respect, digitalisation may also contribute towards mitigating disadvantages of rural areas, fight depopulation, and help reduce spatial inequalities.

Despite these benefits, the adoption of digitalisation also implies major challenges for firms, especially for SMEs, and even more so in rural or peripheral regions. In terms of digital infrastructure, a clear urban/rural divide exists even in highly industrialised countries. Lack of access to high-speed digital infrastructure such as broadband networks may limit the possibilities of digitalisation in rural areas and can contribute towards increasing the inequality between regions (Norris, 2020; Fanelli, 2018). Yet, beyond infrastructure issues, there also exists an urban/rural divide in digital adoption and digital literacy. Salemink et al. (2017) provide a review of the literature on ICT adoption and use in rural areas. They emphasise that digital connectivity could help to overcome the limitations of remoteness of many rural areas, but slower diffusion into rural areas, together with lower levels of skill and education therein, hampers their adoption. Even the acceleration of digitalisation triggered by the Covid-19 pandemic has shown an unequal spatial pattern, with a stronger response in economically more developed locations (Mikhaylova et al., 2021). While most of this literature has focused on household adoption patterns, certain evidence does exist of DT adoption by firms. The literature, for example, has documented higher adoption rates in larger markets and industry agglomerations (Kelley and Helper, 1999; Forman et al., 2005). Fewer studies have, however, specifically focused on SMEs (for a review, see Ramdani et al., 2022). Moreover, the literature has rarely focused on comparisons of DT drivers in SMEs located in different types of locations within the same country (Giotopoulos et al., 2017). The present article strives to contribute to the still emerging literature by comparing drivers of DT adoption between SMEs located in different geographic areas. In this respect, our study is not limited to the analysis of rural-urban differences but also distinguishes between the locations of large and small towns. Furthermore, as shown in Ramandi et al. (2022), most studies to date have focused on rather aggregated ICT information, and evidence on specific DT adoption remains scarce. We contribute to this literature by studying the spatial patterns of drivers for the implementation of new digital technologies in SMEs. Our comparisons of drivers and challenges of different types of DT adoption in different geographic contexts add further insights to the existing knowledge on digitalisation.

In addition, studies on SME digitalisation tend to be based on evidence obtained from case studies and relatively small samples of firms (Fanelli, 2018; Norris, 2020, Aaldering and Song, 2021). Other studies analyse DT and its relationship with the innovativeness of firms at country level (Usai et al., 2021). Although these studies provide important insights into the adoption of digitalisation, they need to be complemented by quantitative analyses of large samples of firms that provide a broader panorama of industries, countries, and different types of locations. This paper contributes to the literature by analysing data provided by the 2020 Flash Eurobarometer 486, which is a statistically representative survey.

The rest of the paper is organised as follows. The “Related Literature” section presents an overview of the related literature. The “Data and stylised facts regarding the adoption of new digital technologies in SMEs” section explains our data set and shows several stylised facts regarding the adoption of digital technologies in European SMEs. The “Econometric analysis” section presents our estimation strategy, and the “Results” section describes the empirical results of the analysis. The “Conclusions” section offers several conclusions and policy implications.

## Related Literature

No homogeneous digital economy exists since the rhythm of adoption of such technologies varies across countries, industries, and between different types of firms and local contexts.

For Europe, there is evidence that many of the strong adopters of DT are in Northern Europe, but Southern Europe also contains major contenders, such as Spain (European Investment Bank, 2021). According to the aforementioned report, in 2020, only 63% of EU firms had adopted at least one digital technology, compared to 73% in the USA. The difference in digital adoption rates between the EU and the USA was particularly significant for small firms (10 to 49 employees). According to the same source, SMEs tend to display lower rates of digital adoption than larger firms. “But the level of adoption for firms with less than 50 employees is particularly low in Europe, where firms tend to be smaller than in the United States” (p. 11). The report concludes that “while large and medium European firms have digitalised almost as fast as their US counterparts, small and micro firms continue to lag behind.”

At the same time, there is evidence of uneven rhythms of DT adoption across industries within the same country. Certain authors believe that their “applicability across the industrial spectrum is unclear” (Buer et al., 2021). A study on OECD countries and large non-OECD countries finds that the most digital intensive sectors are Knowledge-intensive businesses (KIBs) and transport equipment (Calvino et al., 2018). On analysing start-ups in several European process industries, Aaldering & Song (2021) find that certain industries that they term as “conservative” (e.g., wood processing and paper and chemical industries) show a slower rhythm of adoption while biotechnology, pharmaceutical, and food and beverage display a quicker flow. Country specialisation can play a major role. According to Hwang & Kim (2021), in Korea, manufacturing SMEs active in non-metallic mineral products and in basic metals and fabricated metal products are particularly eager to adopt DT.

A powerful motive for adopting digitalisation technologies is triggered by the need to establish close relationships with other actors of the value-chain. For instance, process industries, such as the chemistry or pharmaceutical industries, are dependent on close collaboration along the supply chain (Blichfeldt & Faullant, 2021). On analysing a sample of large domestic firms that operate in the Brazilian food and beverage industry, one study finds that the percentage of adopters of DT clearly increases when dealing with technologies used for coordination with suppliers (Rama & Wilkinson, 2019). The aforementioned authors suggest that a powerful objective for adopting this technology may be the traceability of products across the food chain. On analysing an Italian rural area, Fanelli (2018) finds that SMEs in catering services and in sectors such as information technology, water management, and construction, aimed to expand their respective markets and, in so doing, chose to implement e-platforms for product promotion and exports, online orders and delivery tools, and food traceability rather than investing in R&D activities.

The literature also points to differences in the predisposition of different types of firms to adopt DT, with size constituting a predominant consideration (Acs and Audretsch, 1990; Giunta and Trivieri, 2007; Denicolai et al., 2021; EIB, 2021). While small firms are undoubtedly important for generating technological change (Acs and Audretsch, 1990), they also face barriers, such as lack of internal resources, and experience greater difficulties in appropriating returns from investing in new technology. Several technology adoption studies have documented higher adoption rates among larger firms (Giunta and Trivieri, 2007; Buer et al., 2021; Holl & Mariotti, 2021). Lucchetti & Sterlacchini (2004), who analysed SMEs in Ancona (Italy), found that size did affect the adoption of marketing-oriented DT but not that of e-mail, the Internet, and production-integrating ICT, such as robotics. An analysis of Norwegian manufacturing firms observe that large enterprises have a significantly higher level of shop floor digitalisation and organisational IT competences than do SMEs (Buer et al., 2021). However, on analysing SMEs in three Visegrad Group countries, Vavrecka et al. (2021) found that adoption of marketing-related DT, such as SMS campaigns, did not depend on the size of the firm.

Audretsch et al. (2020) emphasise the importance of innovative start-ups for technological change. In the example of the emergence of digital platform economies, Acs et al. (2021) show the major role that the entry of new firms played for the introduction of the new technologies. Aaldering & Song (2021) claim that start-ups are more likely to adopt digitalisation technologies than incumbent companies. In their view, start-ups are drivers of digitalisation in the European process industries.

Fanelli (2018), Aaldering & Song (2021), and Blichfeldt & Faullant (2021) find that the adoption of DT may be related to the innovativeness of the firm, although it is unclear whether a trade-off between adopting DT and performing R&D does exist. Holl & Mariotti (2021) show that the adoption of logistics innovations, including the adoption of new digital technologies and processes is strongly related to product innovation by the firm. Blichfeldt & Faullant (2021) argue that the companies with higher breadth and depth of digital adoption tend to be highly innovative concerning both new products and new services. A review of the literature suggests that manufacturers adopt DT in the belief that this strategy will help them to trigger different types of innovations (Yang et al., 2021). In contrast, on analysing EU countries, Usai et al. (2021) find only a weak correlation between digitalisation and the innovative performance of firms and, on studying Tunisian SMEs, Kossaï et al. (2020) find none between digitalisation and the importance of R&D activities.

As noted by Acs et al. (2021: p.9), the “platform-based ecosystem is immediately global in nature”; it is developed not by regions and/or national governments, but instead by platform organisations. Despite a “spaceless” nature of many DT technologies, the local and regional environment still matters for technology diffusion and adoption; primarily through knowledge spillovers that influence the learning about new technologies, and through differences in skill levels required for successful implementation. The literature on information and communication technology (ICT) has hence shown that adoption rates are higher in larger markets (Kelley and Helper, 1999; Forman et al., 2005). Regarding the digitalisation of manufacturing processes, there is also empirical evidence that adoption is positively related to industry concentration (Kelley and Helper, 1999), the number of prior adopters (No, 2008) in the firms’ environment, and city size (Holl et al., 2013). Dyba et al. (2022) observe different levels of preparedness for Industry 4.0 adoption in European regions in terms of the presence of broadband connectivity, high skill levels, and substantial R&D expenditure. The literature on the rural-urban digital divide aims to explain the digital inequalities experienced by rural communities (Norris, 2020). Certain studies have pointed to the specific difficulties facing rural communities and small towns. Guzhavina (2021) studies small towns located in a Russian region and notes certain difficulties of DT implementation owing to insufficient local capabilities. Fanelli (2018) concludes that the difficulties facing SMEs in rural areas include limited access to high-speed and affordable Internet, the high cost of online platforms, lack of a secure payment system, the absence of a human interface in e-commerce, and lack of information on traceability systems. In her analysis of Molise (Italy), the aforementioned author observes difficulties, such as those of the characteristics of the business environment itself: the small size of the local market, limited opportunities for trade and networking with other local businesses, and a restricted skill base of the local labour market. Other authors emphasise the need to move the debate beyond broadband connectivity and the urban-rural divide (Cowie et al., 2020). Moreover, the aforementioned authors maintain that the impacts of Industry 4.0 technologies could be just as important in rural as in urban places, although drivers and barriers may differ. In analysing data for China, Shao et al. (2021) confirm this point of view. They find that, concerning jobs and employability of residents, the introduction of the Internet generates greater benefits in rural areas than it does in urban areas. Cowie et al. (2020) conclude that digitalisation has been implemented mainly to face the problems of urban areas and not those of rural areas, such as poor connectivity. On the other hand, they claim, most of the debate on digitalisation in rural areas focuses on agriculture; hence, our interest lies in focusing on rural SMEs that are active in sectors other than agriculture.

Most studies analyse each type of area in isolation or offer analyses of specific regions or cities, and comparative analyses remain scarce. The contribution of the present study includes the comparison of different types of areas (large towns, small towns, rural areas, and other areas) across 34 European countries.

## Data and Stylised Facts Regarding the Adoption of New Digital Technologies in SMEs

### Data

We use data from the Flash Eurobarometer 486 “SMEs, Start-ups, Scale-ups, and Entrepreneurship” conducted on behalf of the European Commission. This survey was carried out between 19th February and 5th May 2020 in 27 EU countries plus the UK and 6 other non-EU European countries, as well as in 5 non-European countries. The target population involved firms with 1 to 250 employees. The aim of the survey was to collect information on barriers and challenges for European SMEs’ related to growth, sustainability, and digitalisation.

Regarding digitalisation, firms were asked about the adoption of the following seven digital technologies:

1. Artificial intelligence (e.g., machine learning and technologies that identify objects or people)
2. Cloud computing (i.e., storing and processing files or data on remote servers hosted on the Internet)
3. Robotics (i.e., robots utilised to automate processes in construction, design, etc.)
4. Smart devices (e.g., smart sensors and smart thermostats)
5. Big data analytics (e.g., data mining and predictive analysis)
6. High-speed infrastructure
7. Blockchains

In addition, firms were also asked whether they had any interest at all in digitalisation. Our analysis is restricted to the 34 European countries. Table 6 in the Appendix  (column 1) shows the composition of our sample in terms of countries.

### Some Descriptive Statistics and Stylised Facts

Table 1 indicates the level of adoption of the seven different types of DTs in our sample. Approximately 68% of the total number of firms responded that they had adopted DT, and only about 4% reported that they were not interested in DT. A high percentage of adopters concerning cloud computing (48% of firms) and high-speed infrastructure (33%) is especially noticeable. Blockchains and AI are the technologies that have been adopted the least.

**Table 1:** Digital technology adoption

	Mean adoption rate (%)
Adoption:	
Any of the following 7 digital technologies	67.5
Artificial intelligence	7.2
Cloud computing	48.0
Robotics	8.7
Smart devices	27.1
Big data	14.2
High-speed infrastructure	33.1
Blockchains	3.1
None of the 7 digital technologies	32.5
Firm has no interest in digitalisation	4.3

Data source: Flash Eurobarometer 486. Authors' own calculations

The degree of diffusion of DT also varies, however, by sector. The Flash Eurobarometer 486 provides the sector of the firm aggregated at the section level. Based on this information, the mining-water-electricity sector, the manufacturing sector, and the construction sectors can be distinguished. For services, the knowledge-intensive service classification is employed based on the two-digit level. This allows us to identify services that are less knowledge-intensive, services sectors with mixed knowledge intensity, and knowledge-intensive services (KIS) (Table 2). Row 1 of Table 2, which displays the percentages of adopters of any of the seven technologies studied herein, shows that DTs are more widely adopted by firms active in KIS and manufacturing, while those active in construction and low-tech services display the lowest levels of adoption in the sample. Differences are also clear regarding the degree of adoption of each of the seven different technologies (Table 2, rows 2–8). For instance, manufacturing firms are more likely to have adopted robotics (20% of companies) than firms in other sectors. By the same token, KIS firms are more likely to have adopted cloud computing (61%) and high-speed infrastructures (41%).

**Table 2:** Digital technology adoption by sector and type of technology (% of SMEs)

	Mining water and electricity	Manufacturing	Construction	Less knowledge-intensive services	Mixed services	KIS services
Adoption:						
Any of the 7 digital technologies	66.5	68.8	60.0	63.6	65.1	76.7
Artificial intelligence	5.1	8.0	3.8	5.3	5.7	11.8
Cloud computing	46.3	44.9	41.0	43.4	46.4	61.1
Robotics	8.6	20.1	4.4	5.6	3.8	7.8
Smart devices	39.7	32.8	23.8	24.7	25.5	27.4
Big data	20.6	13.8	6.2	13.3	13.0	19.2
High-speed infrastructure	26.4	30.3	25.5	32.5	30.0	41.4
Blockchains	3.1	3.6	1.4	2.7	2.9	4.2
None of the 7 digital technologies	33.4	31.2	40.0	36.4	34.9	23.3
Firm has no interest in digitalisation	1.6	3.8	6.8	5.2	5.5	1.9
No. of observations	257	2729	1364	5058	1461	3342

Data source: Flash Eurobarometer 486. Authors’ own calculations

In Table 6 of the Appendix, column 2 shows the percentages of adopters of any DT in the EU and in other European countries. Substantial variations can be observed, with the highest percentages of adopters to be found in Iceland, Sweden, Norway, and the Netherlands. In contrast, the firms more inclined to declare that they have no interest in DT are those located in the Baltic countries (column 10). Differences in the types of DT (columns 3–9 of Appendix Table 6) adopted by SMEs located in the different countries are clear and probably depend on distinct national production structures. Spain, for instance, is not a leading country concerning DT adoption; however, it does display one of the highest percentages of adopters for, specifically, robotics, probably due to its strong position as an exporter of cars and machine tools.

Regarding within-country location characteristics, the Flash Eurobarometer 486 asked firms to state in which of the following areas the enterprise is located:

1. In a large town or city
2. In a small town or village
3. In a rural area
4. In an industrial area
5. Near a border with an EU country
6. Near a border with a non-EU country

The question permitted multiple responses, except for options 1 and 2 which could not be selected simultaneously. Nevertheless, two companies stated that they were in a large town and in a small town; in cleaning the data set, these two observations were dropped. For the remaining firms, six dummy variables were prepared, where a further five companies were dropped for responding that they were located in a large town and in a rural area, and nine companies that responded being located in a small town and in a rural area at the same time. This left us with three exclusive groups of firm location in large towns, small towns, and rural areas. In the sample, 649 companies responded as neither being located in a large town, nor in a small town, nor in a rural area. These companies are classified under being located in other types of locations.

The distribution of firms according to the different types of locations is shown in Table 3. Starting with the non-exclusive location types, firms located in industrial areas are the most likely to have adopted DT and the least likely to be uninterested in such technologies. This is followed by firms located in border areas of the EU. These SMEs probably implement DT because their location may favour involvement in exports and transnational projects. As for our three exclusive location types, adoption is highest in large towns, followed by smaller towns, and firms located in rural areas are the least prone to adopt DT and the most likely to be uninterested in such technologies.

**Table 3:** Digital technology adoption by type of location and type of technology (% of SMEs)

	No. of observations	Any digital technology	Artificial intelligence	Cloud computing	Robotics	Smart devices	Big data	High-speed infrastructure	Blockchains	No digital innovation	No interest
*Exclusive location types*											
Large town	6850	70.1	8.0	51.4	8.4	26.9	17.2	36.8	3.8	29.3	4.3
Small town	5209	64.4	6.7	44.3	8.6	26.9	11.2	30.1	2.3	35.6	4.1
Rural area	1440	62.1	5.8	42.9	8.5	27.4	9.4	25.4	2.2	37.9	5.0
Other	649	72.1	6.8	51.1	12.2	31.3	16.5	36.1	5.1	27.9	4.1
*Non-exclusive location types*											
Industrial area	1722	77.2	8.8	59.0	14.7	34.6	17.0	39.1	4.1	22.8	3.3
EU-border	1293	71.1	8.0	52.0	10.4	31.5	16.8	34.5	4.2	28.8	3.9
Non-EU border	369	65.6	8.1	51.2	10.6	26.6	18.7	29.0	1.9	34.4	3.3

Data source: Flash Eurobarometer 486. Authors’ own calculations

The Flash Eurobarometer 486 also includes a question on how firms rate their regional business environment on a 4-point Likert scale in terms of the following:

1. Overall strength and performance of the regional business environment
2. Access to and collaboration with business partners, such as other enterprises, the public sector, educational institutions, and research organisations
3. Availability of staff with the right skills, including managerial skills
4. Infrastructure for businesses, such as available office space and Internet connectivity

Table 4 shows the differences in the ratings of their regional business environment between firms that have adopted any of the seven digital technologies and firms that have adopted none thereof. Pearson’s Chi^2^ tests are utilised to test for the association between the above characteristics of the environment and whether the firm is a DT adopter. The association is always positive and statistically significant. These results suggest that a good local environment is a driver of digital adoption in SMEs.

**Table 4:** Ratings of the regional business environment: adopters versus non-adopters

	% Very good	% Fairly good	% Fairly poor	% Very poor	Pearson chi^2^: independence between non-adopters and adopters
*Overall strength and performance*					
Non-adopters	15.6	62.4	17.1	4.9	***
Adopters	21.7	63.2	12.2	2.9	
*Collaboration with business partners*					
Non-adopters	16.2	62.2	16.3	5.3	***
Adopters	21	62.1	13.6	3.3	
*Skills*					
Non-adopters	14.1	46.1	28.1	11.8	***
Adopters	15.8	44.3	30	9.9	
*Infrastructure*					
Non-adopters	28.1	58.6	10.3	3	***
Adopters	37.7	51.7	8.5	2.3	

Data source: Flash Eurobarometer 486. Authors’ own calculations

*Significance at the 10% level, **5% level, and ***1% level

## Econometric Analysis

We estimate the probability that a SME has adopted digital innovations. The firm adopts a new digital technology if the anticipated benefits of adoption exceed the cost. However, only the adoption or non-adoption of technology *τ* is observed.

Adoption *y*_*iτ*_ of firm *i* of technology *τ*, *τ*=1,2 is then captured by a binary choice model

1 $${y}_{i\tau}=\left\{\begin{array}{c}1\ \textrm{if}\ {y}_{i\tau}\ge 0\\ {}0\ \textrm{else}\end{array}\right.$$

where the latent variable ${y}_{i\tau}^{\ast }$, representing firm *i*’s net value from adopting the new technology *τ*, is a linear function of observable firm-specific characteristics *c*_*i*_, industry characteristics *p*_*i*_, and location characteristics *r*_*i*_, and where *ν*_*iτ*_ is a standard normal term.

2 $${y}_{i\tau}={c}_i{\beta}_{1\tau }+{p}_i{\beta}_{2\tau }+{r}_i{\beta}_{3\tau }+{\nu}_{i\tau}$$

Our key variables of interest refer to the characteristics of the location of a firm. In our estimations, we test for the influence of large town location compared to small town location, rural location, and other locations on the probability that a firm has introduced digital technologies, where the group of “other” locations includes all those firms that have neither responded that they are located in a large town, in a small town, or in a rural location.

Our control variables of the model are selected in accordance with the indications taken from previous studies on DT adoption. Based on the literature reviewed in the “Related Literature” section, the variable *size*, measured in terms of the number of employees (in logs), is included to control for size differences between SMEs. The variable *age*, calculated as the current year minus the year the company was first registered (in logs), is also included to capture newly created firms. In order to capture the degree of innovativeness of the firm and to distinguish between the different types of innovation, dummies are included for green innovation (*green_inno*), product innovation (*prod_inno*), process innovation (*proc_inno*), management innovation (*manag_inno*), and for marketing innovation (*sales_inno*).

Ownership may also influence the likelihood that a firm adopts digitalisation. Small and medium-sized firms that do not belong to a business group may face financial and technical difficulties when attempting to adopt new technology. In a Hungarian region, foreign multinational enterprises (MNEs) seem more likely than domestic firms to adopt Industry 4.0 (Nagy et al., 2020). We include the variable *group* which takes value 1 for firms that are part of a national or international group, and zero otherwise.

Furthermore, high-growth enterprises have been found to present a higher propensity for adoption of DT than other firms (Giunta and Trivieri, 2007; Benedetti Fasil et al., 2021). The variable *growth* is included, which is based on the Likert variable for turnover growth since 2016.

There is also evidence that a firm’s export status is of importance. Giunta and Trivieri (2007), for example, find that firms that export show a greater probability for IT adoption. Teruel et al. (2021) show that firms that adopt new DT have a higher probability of being internationalised, especially via exports. In a sample of Tunisian SMEs, export (and import) intensities are associated to ICT adoption (Kossaï et al., 2020). The dummy variable *non-export* is included, which takes value 1 if the firm reports having sales only in its domestic market, and zero otherwise.

As indicated by our descriptive statistics and also in certain recent empirical studies, the various services and industrial sectors seem to display different rhythms of adoption (Rama & Wilkinson, 2019; Aaldering & Song, 2021; Hwang & Kim, 2021). Sectoral dummies for 16 sectors are therefore included based on the section level information provided in the survey.

In order to control for country-specific differences, country dummy variables are also included.

## Results

Column 1 of Table 5 shows the estimation results for the probability that a company has introduced any of the surveyed digital technology, while columns 2 to 8 estimate whether the company has introduced specific types of advanced DT. Column 9 assesses the probability that a company declares no interest in DT. The reported coefficients in all the columns are the marginal effects.

**Table 5:** Probit estimation results: marginal effects

Variables	(1)	(2)	(3)	(4)	(5)	(6)	(7)	(8)	(9)
	digi_inno	digi_AI	digi_cloud	digi_robot	digi_smart	digi_big	digi_infra	digi_block	digi_no_interest
*smalltown*	−0.033***	−0.003	−0.048***	−0.005	−0.000	−0.030***	−0.031***	−0.011***	0.001
	(0.008)	(0.005)	(0.009)	(0.005)	(0.008)	(0.007)	(0.009)	(0.004)	(0.004)
*rural*	−0.068***	−0.015*	−0.074***	−0.015*	−0.006	−0.061***	−0.092***	−0.012**	0.006
	(0.013)	(0.008)	(0.014)	(0.008)	(0.013)	(0.011)	(0.014)	(0.006)	(0.006)
*other location*	−0.006	−0.030***	−0.010	0.006	0.006	−0.021	−0.042**	0.004	0.011
	(0.019)	(0.012)	(0.020)	(0.010)	(0.018)	(0.014)	(0.019)	(0.007)	(0.009)
*size (log)*	0.043***	0.014***	0.028***	0.023***	0.040***	0.033***	0.030***	0.006***	−0.008***
	(0.003)	(0.001)	(0.003)	(0.002)	(0.003)	(0.002)	(0.003)	(0.001)	(0.001)
*age (log)*	−0.003	−0.007**	−0.003	−0.003	−0.005	−0.017***	−0.003	−0.009***	−0.000
	(0.006)	(0.003)	(0.006)	(0.003)	(0.005)	(0.004)	(0.005)	(0.002)	(0.003)
*growth*	0.026***	0.004*	0.033***	0.006**	0.015***	0.007**	0.009**	0.001	−0.005***
	(0.004)	(0.002)	(0.004)	(0.002)	(0.004)	(0.003)	(0.004)	(0.002)	(0.002)
*group*	0.083***	0.014**	0.072***	0.032***	0.004	0.044***	0.042***	0.002	−0.022**
	(0.016)	(0.007)	(0.015)	(0.007)	(0.013)	(0.009)	(0.013)	(0.005)	(0.009)
*non_export*	−0.088***	−0.031***	−0.077***	−0.035***	−0.036***	−0.033***	−0.057***	−0.007**	0.013***
	(0.009)	(0.005)	(0.009)	(0.005)	(0.008)	(0.006)	(0.009)	(0.003)	(0.004)
*green_inno*	0.104***	0.031***	0.049***	0.027***	0.134***	0.051***	0.060***	0.015***	−0.011**
	(0.010)	(0.005)	(0.010)	(0.005)	(0.009)	(0.007)	(0.009)	(0.003)	(0.005)
*prod_inno*	0.061***	0.020***	0.060***	0.019***	0.062***	0.036***	0.044***	0.010***	−0.019***
	(0.009)	(0.005)	(0.010)	(0.005)	(0.009)	(0.006)	(0.009)	(0.003)	(0.005)
*proc_inno*	0.069***	0.023***	0.040***	0.051***	0.058***	0.027***	0.044***	0.007*	−0.026***
	(0.011)	(0.005)	(0.011)	(0.005)	(0.009)	(0.007)	(0.010)	(0.004)	(0.006)
*manag_inno*	0.077***	0.012**	0.077***	0.008	0.034***	0.040***	0.028***	0.011***	−0.013**
	(0.012)	(0.006)	(0.012)	(0.006)	(0.010)	(0.007)	(0.011)	(0.004)	(0.007)
*sales_inno*	0.068***	0.012**	0.054***	−0.002	0.027***	0.036***	0.058***	0.007*	−0.027***
	(0.010)	(0.005)	(0.011)	(0.006)	(0.009)	(0.007)	(0.010)	(0.004)	(0.006)
Observations	12,915	12,915	12,915	12,915	12,915	12,915	12,915	12,723	12,776
Pseudo_R2	0.167	0.144	0.145	0.207	0.110	0.152	0.160	0.108	0.126
LL	−6755	−2925	−7648	−3061	−6721	−4454	−6906	−1586	−2014

Standard errors in parentheses

***p<0.01, **p<0.05, *p<0.1

The *smalltown* variable displays a negative and significant coefficient, which indicates that compared to location in a large town, location in a small town decreases the probability that a firm has adopted DT. The probability for DT adoption (independent of its type) decreases by −3.3%. This shows that firms in small towns have a higher probability of not having adopted DT. The *smalltown* coefficient is also negative and significant for *digi_cloud*, *digi_big*, *digi_infra,* and *digi_block*. The coefficients of the variables for *digi_AI*, *digi_robot*, and *digi_smart* are also negative but they are not statistically significant.

The coefficient of the *rural* variable is also negative and statistically significant for *digi_inno* and, specifically, for each type of DT; the exception is *digi_smart* since in this case the coefficient is negative but not statistically significant. For all specifications, marginal effects are greater than those concerning *smalltown.* Compared to location in a large town, location in a rural area decreases the probability that a firm has adopted any type of DT by almost −7%. The reduction is still greater regarding *digi_infra* (−9.2%). On the other hand, compared with *smalltown*, the breadth of technology adoption in rural areas is clearly more limited.

The coefficients of the *other location* variable are, in most cases, not statistically significant, with two exceptions: they are negative and statistically significant concerning *digi_AI* and *digi_infra*. Compared to a location in a large town, location in *other areas* decreases the probability that a company has adopted *digi_AI* by −3% and the probability that it has adopted *digi_infra* by −4.2%.

Column 9 shows the results for the probability that a firm declares that it has no interest in digitalisation. None of the location variables is now significant. This shows that while there are significant differences in the probability of DT adoption across different types of locations, this does not hold for their declared interest, once country, sector, and firm-specific differences are controlled for. Note, however, that declared interest is significantly related to most firm-specific characteristics. Hence, the higher proportion of firms with no interest in digitalisation that is observed for smaller towns and especially for rural areas is principally due the types of firms located in those areas.

Regarding the specific characteristics of the firms, our results show that the coefficient of the *size* variable, which measures the size of the company in terms of their number of employees, is always both positively and statistically significantly related to DT adoption. For DT adoption, in general, a relatively larger size of SMEs increases the probability of DT adoption by 4.3%. In our sample of SMEs, this shows that medium-sized firms tend to be more prone to adopting DT than are smaller firms, and these differences are substantial. Our results do not support those of Vavrecka et al. (2021) concerning SMEs and their patterns of adoption of marketing-related DT nor does it support those of Lucchetti & Sterlacchini (2004) related to different effects of size on adoption of different types of ICT. In our sample, size is always significantly associated to DT adoption. In contrast, our results support those of Giunta & Trivieri (2007), Buer et al. (2021), and Hizam-Hanafiah & Soomro (2021) in that size of the company appears to be positively associated with the adoption of DT. Holl & Mariotti (2021), in their analysis of German companies, also find that logistics innovation relating to DT tends to be adopted by larger firms. However, our result needs to be taken with caution since our sample is limited to SMEs.

As for *age*, the estimated coefficients are negative, and although not always statistically significant, they do indicate a tendency for younger SMEs to exhibit a higher propensity to adopt DT. However, for digital technology adoption by SMEs in general (*digi_inno*), our results seem not to support the clear leading role of start-ups in driving digitalisation as suggested in several previous studies (Audretsch et al., 2020; Acs et al., 2021; Aaldering & Song, 2021). Nevertheless, our results do show that, “younger” firms seem more prone than incumbents to adopting specific DT, such as AI, Big data, and blockchain technology. This is an interesting finding and in line with recent studies (Obschonka & Audretsch, 2020; Fossen & Sorgner, 2021) that suggest that new disruptive technologies, such as AI and Big data, can possess the potential to generate new digital entrepreneurial activity.

Small and medium-sized enterprises (SMEs) that display a rapid rhythm of growth, those that export, and those that belong to business groups, are also more likely to be adopters than, respectively, independent SMEs, SMEs that display slow growth, and non-exporters. Concerning exporters, our results support those of Giunta and Trivieri (2007) and Teruel et al. (2021) in that internationalisation goes hand in hand with the adoption of DT. Our results regarding expanding firms are similar to those found by Benedetti Fasil et al. (2021). Finally, the support of a business group seems to be important to encourage a SME to adopt DT. Group ownership increases by 8% the probability of DT adoption. Interestingly, these firm-specific characteristics are also significantly related to firms’ interest in digitalisation. Firm growth, belonging to a group and being engaged in international markets may generate a greater need for digital solutions.

Our results further show that innovators are always more likely to adopt DT than non-innovators, and this applies to all types of innovation, but especially to green innovation and management innovation. Involvement in green innovation increases the probability that a firm has adopted any kind of DT by 10.4% (and by 13.4% adoption of *digi_smart*). Involvement in management innovation increases the probability of DT adoption by nearly 8%. Conversely, innovators are less likely to declare that they have not adopted DT or that they are not interested in such technologies. Our results support those of Blichfeldt & Faullant (2021). We found no trade-off between the propensity of the firm to adopt DT and the performance of innovations. Nevertheless, this could occur in specific circumstances, as suggested by Fanelli (2018). On the other hand, our results also highlight that firms with no interest in digitalisation are also clearly less innovative in general.

Due to size limitations, coefficients for the marginal effects of the country dummy variables are not shown in Table 5, but instead in Table 7 in the Appendix. With France taken as the base country, we observe that location of a company in the Netherlands, Sweden, Norway, and Iceland increases its chances of DT adoption versus those of a company located in France. This confirms the findings presented in the descriptive statistics. One may conclude that these countries tend to lead the diffusion of DT in Western Europe, even when variables denoting characteristics of the firm, such as innovativeness, are taken into account; probably due to a greater capability of leveraging the advantages of new digital technology (Tranos, 2012). Similarly, due to size limitations, the sector dummy variables are not shown either in Table 5. 1 Taking mining as the base sector for comparisons, companies active in ICT, financial services, and professional and scientific services are more likely to utilise DT than are firms in the mining sector. In contrast, companies active in construction, wholesale, and accommodation are more likely to declare that they are not interested in DT than are those active in the Mining sector. Despite the use of different methodologies, our results are in accordance with those of Calvino et al. (2018).

## Conclusions

We have striven to understand the determinants of digital technology (DT) adoption in small and medium-sized enterprises (SMEs) of the European Union, with a special interest in the possible influence of location. Our article contributes towards the debate on location and technological diffusion with the analysis of a statistically representative sample of firms active in a variety of countries and sectors.

Our analysis shows considerable heterogeneity of SMEs in their propensity to adopt advanced digital technologies. Independently of geographic location, the probability of DT adoption is always positively associated to the SME being in a good local business environment, with a pool of possible partnerships, availability of skilled workers, and business infrastructure. Business environments seem to be major drivers of adoption, whatever the location of the SME.

Significant differences are also found in the likelihood of the adoption of digital technology of SMEs between large towns, small towns, and rural locations. Firms located in large towns display the most significant depth and breadth of DT adoption even when country, sector, and specific characteristics of firms, such as group membership, are controlled for. In this respect, they are followed by firms located in small towns. In contrast, SMEs located in rural areas are the least likely to be adopters. The probability of adoption clearly decreases the less dense the agglomerations are. These results highlight that the urban-rural digital divide also constitutes a major challenge for the adoption of advanced digital technology by SMEs. However, regarding differences in the interest expressed by firms regarding digitalisation, our results show that such differences are primarily due to specific characteristics of the firm rather than to location in itself.

Size, firm growth, pertaining to a group, exporting, and innovativeness are all strongly associated with DT adoption. SMEs do not seem to be substituting DT adoption with innovation; rather, they employ adoption and innovation as complementary strategies. We also find evidence for digital entrepreneurship related to AI, Big data, and blockchains. Our results further suggest the need for a specific analysis of independent SMEs, given the key importance of ownership. This certainly provides an avenue for future research.

Our analysis should also be informative for policy-makers that aim to provide incentives to accelerate digital transformation by providing findings of a more nuanced nature that can help in designing tailored policies. This is particularly relevant in the fight against the urban-rural divide, in making growth more inclusive, and in the reduction of spatial inequalities. Our study highlights the importance of focusing on small rural businesses that are active in sectors other than agriculture, a question often neglected by both policy-makers and scholars. The digital weakness of such companies is evident concerning all the spectrum of DT but, especially, regarding high-speed infrastructure. Yet, digital infrastructure provision on its own will remain insufficient. Rural digital hubs could, in addition to infrastructure, provide support to SMEs in the digitalisation process, but their successful implementation is also facing specific constraints (Rundel and Salemink, 2021).

In fact, our study shows that the European SMEs that are least interested in digitalisation are also those located in rural areas. One possible reason may involve the lack of knowledge regarding digitalisation, particularly about the more advanced applications, and insufficient information on the applied benefits of such technologies. As argued in Yu and Schweisfurth (2020), knowledge of the technology and its benefits constitutes a key factor in the adoption process of new technologies and a major barrier for SMEs. Consequently, we suggest that DT adoption is promoted not in isolation but in conjunction with other programmes (i.e., management upgrading programmes) so that the entrepreneurs and their staff can understand the practical interest of such technologies. An approach focusing on the whole value-chain is essential: for instance, digitalised traceability systems, which include suppliers, manufacturers, service firms, and clients. Policies could also be geared towards stimulating knowledge transfers between SMEs and technology producers or existing advanced users of digital technologies (Yu and Schweisfurth, 2020).

Our study also reveals the strong association between digitalisation and innovation, “green” innovation being an especially important driver of DT adoption. The presence, in rural areas, of manufacturing SMEs devoted to the processing of agricultural inputs, opens the way to the support of “green” innovation in aspects such as rational use of water, energy saving, and utilisation of agricultural sub-products, which are often wasted. This strategy, in turn, may contribute to the digitalisation of rural SMEs.

## Appendix

See Tables 6 and 7.

**Table 6:** Digital technology adoption per country and type of technology (% of SMEs)

	No. of observations	Any digital technologies	Artificial intelligence	Cloud computing	Robotics	Smart devices	Big data	High-speed infrastructure	Blockchains	No interest
France	501	76.0	8.2	48.5	11.0	19.0	10.0	55.3	3.5	4.0
Belgium	495	75.6	9.1	59.8	10.1	31.7	16.2	30.0	2.6	3.0
Netherlands	497	86.3	12.3	71.8	12.7	42.7	23.9	58.1	0.8	0.4
Germany	498	75.7	0.8	51.6	9.2	29.9	9.8	44.0	2.6	1.6
Italy	480	37.5	4.0	24.8	3.8	10.2	5.0	11.7	2.1	4.5
Luxembourg	197	81.2	17.8	55.3	8.1	29.4	19.8	64.0	7.6	3.0
Denmark	497	75.7	10.1	63.0	14.7	24.3	22.5	33.6	2.0	7.0
Ireland	497	78.1	10.1	61.2	7.0	44.1	17.7	46.9	5.4	0.8
UK	495	74.1	7.1	58.4	4.8	34.1	15.4	34.5	2.6	1.4
Greece	497	72.2	4.6	44.9	4.2	22.9	19.7	46.5	5.6	3.4
Spain	500	78.4	11.4	59.6	14.6	30.8	16.4	51.0	5.2	1.2
Portugal	495	68.5	7.9	51.7	8.7	24.2	10.3	39.4	4.2	2.6
Finland	495	74.1	12.9	64.4	14.3	30.9	19.2	30.3	2.4	2.8
Sweden	496	85.3	11.9	74.0	13.5	31.7	18.5	38.1	2.6	3.5
Austria	491	69.5	9.6	47.0	10.8	29.7	15.7	37.7	4.9	3.0
Cyprus (Republic)	199	79.9	7.0	45.7	7.0	24.1	21.1	60.8	3.0	8.7
Czech Republic	498	65.2	5.8	45.4	7.2	28.3	14.5	32.5	1.8	3.0
Estonia	494	66.1	2.4	44.3	8.3	22.3	7.9	43.9	1.4	12.8
Hungary	487	63.4	1.2	36.6	5.3	33.1	3.5	18.4	6.1	6.6
Latvia	492	65.2	5.1	51.4	9.8	25.6	16.7	32.1	4.3	9.5
Lithuania	491	43.4	1.6	30.1	4.1	17.7	6.1	4.9	1.0	9.4
Malta	199	62.3	6.5	43.7	7.0	27.6	13.1	31.7	4.5	3.6
Poland	497	53.7	6.0	31.0	9.3	27.4	17.5	11.5	2.2	2.6
Slovakia	484	53.7	5.6	28.9	7.9	22.7	10.3	21.5	1.9	5.5
Slovenia	498	70.9	6.2	55.4	10.0	36.5	10.8	15.9	2.6	3.0
Bulgaria	486	56.2	4.1	39.7	7.0	23.7	13.0	18.9	2.3	4.6
Romania	478	47.3	5.0	18.2	7.3	17.6	10.3	22.4	3.3	6.9
Croatia	494	61.5	4.0	43.1	3.8	21.1	11.5	21.5	3.8	2.4
Macedonia/FRYOM	200	53.5	3.5	40.5	5.5	24.0	13.0	7.5	2.0	12.5
Serbia	197	41.1	2.0	23.4	7.1	20.3	8.1	15.2	1.5	3.5
Norway	299	87.0	14.7	77.6	9.4	32.4	17.4	59.3	1.7	1.7
Iceland	194	93.3	10.3	76.3	11.3	39.2	23.2	82.5	3.1	1.2
Bosnia-Herzegovina	196	56.6	4.1	25.5	4.1	25.0	13.3	10.7	0.0	4.7
Kosovo	197	72.1	10.7	13.2	9.6	8.1	24.9	16.8	2.5	3.7

Data source: Flash Eurobarometer 486; Authors’ own calculations

**Table 7:** Coefficients for country dummies (marginal effects)

Countries	(1)	(2)	(3)	(4)	(5)	(6)	(7)	(8)	(9)
	digi_inno	digi_AI	digi_cloud	digi_robot	digi_smart	digi_big	digi_infra	digi_block	digi_no_interest
Belgium	−0.002	0.008	0.116***	0.012	0.118***	0.060***	−0.251***	−0.007	−0.013
Netherlands	0.073***	0.016	0.188***	0.026	0.192***	0.091***	−0.010	0.036**	−0.037***
Germany	−0.010	−0.009	0.019	0.001	0.097***	−0.005	−0.112***	−0.010	−0.023*
Italy	−0.276***	−0.031*	−0.173***	−0.055***	−0.043*	−0.028	−0.387***	−0.019*	−0.013
Luxembourg	0.009	0.081***	0.019	−0.016	0.075**	0.066**	0.055	0.028	0.000
Denmark	−0.045	−0.009	0.094***	0.033*	0.012	0.091***	−0.250***	−0.018*	0.044***
Ireland	0.014	0.003	0.112***	−0.017	0.218***	0.053**	−0.102***	0.017	−0.035***
UK	0.006	−0.012	0.127***	−0.034*	0.159***	0.048**	−0.192***	−0.004	−0.027**
Greece	−0.032	−0.042***	−0.043	−0.047***	0.057**	0.093***	−0.077**	0.014	−0.010
Spain	0.009	0.007	0.101***	0.024	0.081***	0.045**	−0.041	0.012	−0.026**
Portugal	−0.093***	−0.010	0.016	−0.021	0.028	−0.004	−0.165***	0.001	−0.015
Finland	−0.001	0.032*	0.136***	0.046**	0.123***	0.074***	−0.257***	−0.013	−0.012
Sweden	0.097***	0.016	0.242***	0.017	0.103***	0.068***	−0.176***	−0.011	−0.004
Austria	−0.086***	−0.003	−0.027	−0.003	0.076***	0.043**	−0.185***	0.011	−0.012
Cyprus (Republic)	0.049	−0.012	−0.034	−0.012	0.068*	0.102***	0.032	−0.007	0.041*
Czech Republic	−0.106***	−0.024	−0.044	−0.022	0.081***	0.058***	−0.228***	−0.018	−0.013
Estonia	−0.047*	−0.058***	−0.012	−0.007	0.059**	−0.009	−0.075**	−0.018	0.067***
Hungary	−0.095***	−0.070***	−0.106***	−0.036**	0.166***	−0.052***	−0.340***	−0.027***	0.017
Latvia	−0.105***	−0.039**	0.022	−0.002	0.061**	0.055***	−0.238***	−0.002	0.056***
Lithuania	−0.261***	−0.065***	−0.161***	−0.036**	0.041	−0.022	−0.492***	−0.027***	0.042***
Malta	−0.161***	−0.035*	−0.069	−0.038*	0.083**	0.005	−0.270***	−0.006	0.012
Poland	−0.253***	−0.031*	−0.196***	−0.013	0.052**	0.052***	−0.443***	−0.020*	−0.010
Slovakia	−0.194***	−0.023	−0.159***	−0.014	0.041	0.013	−0.318***	−0.015	0.021
Slovenia	−0.045*	−0.028*	0.067**	−0.002	0.180***	0.013	−0.379***	−0.006	−0.012
Bulgaria	−0.177***	−0.045***	−0.070**	−0.030*	0.049*	0.020	−0.355***	−0.016	0.008
Romania	−0.232***	−0.020	−0.273***	0.000	0.018	0.018	−0.302***	0.006	0.023
Croatia	−0.122***	−0.044***	−0.032	−0.058***	0.050**	0.035*	−0.319***	0.011	−0.019
Macedonia/FRYOM	−0.123***	−0.032	0.007	−0.020	0.105***	0.075**	−0.440***	−0.004	0.046**
Serbia	−0.285***	−0.065***	−0.213***	−0.023	0.043	−0.000	−0.376***	−0.014	−0.013
Norway	0.123***	0.070***	0.294***	0.030	0.154***	0.096***	0.058	−0.015	−0.025**
Iceland	0.155***	−0.006	0.238***	0.009	0.140***	0.071**	0.237***	−0.017	−0.022
Bosnia-Herzegovina	−0.140***	−0.027	−0.190***	−0.035*	0.113***	0.065**	−0.420***		−0.001
Kosovo	0.017	0.062**	−0.295***	0.047*	−0.066**	0.194***	−0.342***	−0.004	−0.016

Significance: ****p*<0.01, ***p*<0.05, **p*<0.1

## Footnotes

1 These unreported results are available upon request.
